# Stable Non-Covalent Co(Salphen)-Based Polymeric Catalyst for Highly Efficient and Selective Oxidation of 2,3,6-Trimethylphenol

**DOI:** 10.3390/polym12051076

**Published:** 2020-05-08

**Authors:** Weijie Zhang, Lingling Hu, He Zhang, Chunyue Pan, Juntao Tang

**Affiliations:** College of Chemistry and Chemical Engineering, Central South University, 932 South Lushan Road, Changsha 410083, China; 162301017@csu.edu.cn (W.Z.); hll7756@csu.edu.cn (L.H.); zhanghe@csu.edu.cn (H.Z.)

**Keywords:** co(salphen), supramolecular polymers, heterogeneous oxidation, molecular oxygen, 2,3,5-Trimethyl-1,4-benzoquinone

## Abstract

Developing highly efficient catalyst systems for phenol–quinone transformation is of great significance in the chemical/biological industries. Herein, we reported a novel heterogenous catalytic system based on Co(Salphen) supramolecular polymers (CSP), which delivered an excellent catalytic performance in the oxidation of 2,3,6-trimethylphenol (TMP) under mild conditions. The CSP were constructed through a simple self-assembled process between BiCo(Salphen) complex and 4,4-dipyridine. By applying BiCo-BiPy1:1 CSP as the catalyst, 2,3,5-trimethyl-1,4-benzoquinone (TMBQ) could be obtained with an excellent conversion (>99%) and selectivity over 99% under mild reaction conditions (30 °C, 0.1 MPa). In addition, it can be recycled at least five times without substantial decline in catalytic activities (conversion and selectivity), suggesting its excellent stability and recyclability. This work may provide guidance on designing and building valuable catalysts for environmentally friendly and cost-effective oxidation reactions.

## 1. Introduction

Vitamin E is a valuable lipid-soluble antioxidant in the body, and its annual demands have exceeded more than 30,000 tons around the world [1]. The key intermediate to synthesize vitamin E is 2,3,5-Trimethyl-1,4-benzoquinone (TMBQ) and is usually obtained from the oxidation of 2,3,6-trimethylphenol (TMP) [2,3,4,5,6]. Traditional methods were usually conducted in the presence of stoichiometric metallic oxidants and strong acid, which unfortunately generated large quantities of oxidized byproducts and harmful wastes [7,8,9,10]. Alternatively, developing cost-effective and environmentally friendly catalytic routes has attracted increased attention. A variety of catalytic systems based on “green” or environmentally benign oxidants such as hydrogen peroxide [6,11,12,13,14,15,16] or tert-butyl hydroperoxide (TBHP) [17,18] have been employed. For example, H_2_O_2_ has been utilized by Kholdeeva and Selvaraj et al. to oxidize TMP by applying the mesoporous titanosilicate catalysts which could selectively afford TMBQ in 99% yield with almost 100% conversion at 353 K for 60 min [15,19]. Recently, Kholdeeva et al. designed a novel heterogeneous reaction pathway (a carbon nanotube-loaded divanadium-substituted γ-Keggin polyoxotungstate) and employed H_2_O_2_ as an oxidant for oxidation of TMP, where TMBQ could be obtained in a quantitative yield [15]. Su et al. developed a metal-free heterogeneous catalytic system for selective oxidation of TMP in the presence of TBHP, where the designed catalysts exhibited excellent conversion (>99%) and high selectivity (>99%) in oxidizing TMP to TMBQ [18].

It should be noted that when considering the environmental impact and atom efficiency, searching for a catalytic system using sustainable molecular oxygen is more promising [20,21,22,23]. Currently, one step oxidation of TMP with molecular oxygen using a copper chloride catalyst has been widely employed for synthesizing TMBQ, which could afford a yield up to 86.5% [24]. To further improve the efficiency, two phase medium (alcohol/water) or cocatalysts including hydroxylamine hydrochloride and lithium chloride were introduced [6]. However, the apparent drawbacks such as the formation of Cl-contaminated byproducts and the requirement for corrosion-resistant apparatus limited their practical implementation. Transition metal coordination compounds have long been known as another type of catalysts for oxidation of substituted phenols to quinones in the presence of molecular oxygen, but they are still suffering from issues including metal leaching, low oxidant utilization efficiency and poor recyclability [2,7,8,9,22]. Thus, developing heterogeneous catalysts which could activate molecular oxygen to selectively oxidize TMP with high efficiency remains highly desirable. To the best of our knowledge, limited studies have been conducted regarding this aspect to date [2]. 

**Scheme 1:**
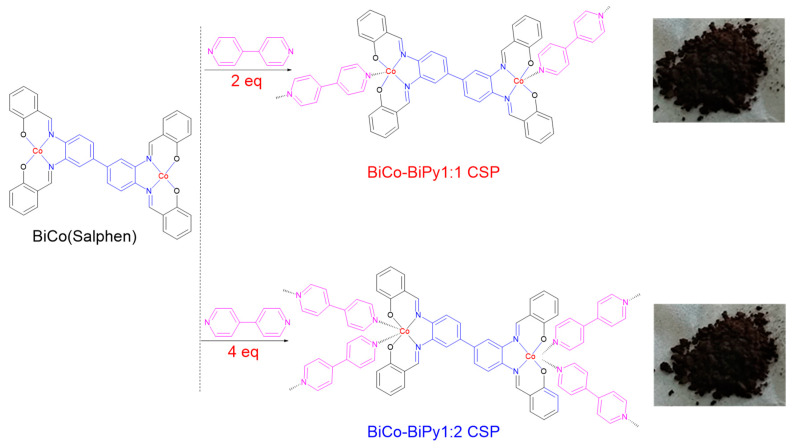
Synthetic routes and structures of supramolecular polymers (BiCo-BiPy1:1 CSP and BiCo-BiPy1:2 CSP).

Motivated by biological systems, supramolecular polymers assembled by non-covalent interactions have attracted increasing attention toward developing them as catalysts for complex transformations in recent decades [25,26]. Compared to polymeric materials connected via strong covalent bonds, they can be conveniently prepared without tedious synthesis. In addition to benefiting from adjustability of the building blocks and active metal centers, metal–supramolecular polymers could also be developed for specific heterogeneous catalytic applications [27,28]. In this work, two supramolecular catalysts (BiCo-BiPy1:1 CSP and BiCo-BiPy1:2 CSP) were constructed from BiCo(Salphen) complex and 4,4-dipyridine (Scheme 1). On one hand, the self-assembled supramolecular catalysts could be readily recovered by simple separation techniques, circumventing inferior ones such as recycling and reusing of homogeneous Salphen complex in practical applications. This is instrumental in reducing the cost of expensive precious metals, especially where the price is greatly affected by market fluctuations. On the other hand, the active catalytic centers were distinctively separated by rigid linkers, and hence, the reactant or substrate accessibility were greatly improved. The selective oxidation of TMP to TMBQ by employing the as-prepared catalysts was evaluated using oxygen as a “green” oxidant under various reaction conditions. Furthermore, the advantages of the supramolecular polymers, in terms of catalytic performance and recyclability over traditional homogenous catalyst and other immobilized ones, have been demonstrated. Compared to homogeneous catalytic systems, the designed Co(Salphen) supramolecular polymer (CSP) catalyst showed promising potentials in industrial application with good recyclability, simplicity (easy operation and separation) and environmental friendliness. We anticipated that this work could provide some guidance on establishing a simple but new design strategy to develop highly efficient catalysts for selective oxidation of phenols.

## 2. Experimental Section

### 2.1. Materials

We purchased 3,3-Diaminobenzidine, salicylaldehyde, 4,4-dipyridine, Co(OAc)_2_·4H_2_O, 1,2-diaminobenzene, pyridine, 2,3,6-trimethylphenol, other solvents and reagents were purchased from Energy Chemical (Shanghai, China) and used them as received without purifications.

### 2.2. Preparation of BiSalphen

To a suspension of 3,3-diaminobenzidine (1.0 g, 4.67 mmol) in methanol (150 mL), the solution of salicylaldehyde (2.74 g, 22.4 mmol) in methanol (100 mL) was added dropwise under N_2_ atmosphere and stirred at 80 °C for 24 h. The resulting precipitate was filtrated and washed with methanol, and then pure product was obtained by recrystallization from hot tetrahydrofuran (THF) in a yield of 91% (4.9 g) and applied in the next step without further purification. ^1^H NMR (CDCl_3_, 400 MHz): δ 13.05–13.00 (d, *J* = 20 Hz, 4H), 8.73–8.71 (d, *J* = 8.0 Hz, 4H), 7.61–7.35 (m, 14H) and 7.07–6.94 (m, 8H) ppm. ^13^C NMR (CDCl_3_, 100 MHz): δ 164.3, 163.5, 161.4, 143.3, 141.8, 139.5, 133.6, 132.5, 132.4, 125.9,120.2, 119.1, 118.4 and 117.6 ppm.

### 2.3. Synthesis of BiCo(Salphen)

To a solution of BiSalphen (251 mg, 0.4 mmol) in THF (100 mL), the solution of Co(OAc)_2_·4H_2_O (238 mg, 0.95mmol) in methanol (30 mL) was added dropwise under N_2_ atmosphere. The mixture was stirred at room temperature for 24 h. The resulting precipitate was filtrated and washed with methanol (30 mL*3), and dried on vacuum to afford BiCo(Salphen) as brown red powder (2.62 g, 89%). A peak at 745.00 corresponding to [M + H]^1+^ for BiCo(Salphen) was detected by high-resolution mass spectrum (HRMS) measurements (Figure S1 in the Supplementary Materials).

### 2.4. Synthesis of BiCo-BiPy1:1 CSP and BiCo-BiPy1:2 CSP 

A suspension of BiCo(Salphen) (100 mg, 0.13 mmol) in *N*,*N*-dimethylformamide (DMF) (150 mL) was stirred at 80 °C for 3 h. Afterward, 4,4-dipyridine (21 mg, 0.13 mmol) was added and stirring of the mixture continued for 48 h. After the mixture was cooled to room temperature, the precipitate was isolated by filtration and extracted with THF and methanol for 24 h, respectively. Finally, the solid was dried at 60 °C for 24 h under vacuum to afford dark brown powder (62 mg, 51%). The synthetic procedure for BiCo-BiPy1:2 CSP was the same as BiCo-BiPy1:1 CSP with the addition of four equivalents of pyridine.

### 2.5. Catalytic Aerobic Oxidation of 2,3,6-Trimethylphenol 

Typical reaction conditions were as follows: The mixture of TMP (68.1 mg, 0.5 mmol), catalysts (10.0 mg) and methanol (2.0 mL) was stirred under O_2_ atmosphere at 30 °C for 12 h. The reaction took place in a high pressure reaction kettle (25 mL) with the oxygen pressure set up at 1.0 MPa. In cycling tests, the catalyst was separated by high-speed centrifugation and washed with methanol, dried and used in the next cycle. The reaction kinetic curves were obtained by five parallel experiments. The products were identified by ^1^H NMR and ^13^C NMR.

### 2.6. Instruments 

^1^H and ^13^C NMR spectra were obtained on Bruker AM-400 MHz (Leiderdorp, The Netherlands). Fourier Transform Infrared spectroscopy (FT-IR) spectra were collected on a V Nicolet 6700 FTIR (Nicolet Co., Nicolet, MN, USA). UV-vis absorption spectra were recorded using a UV 765 spectrophotometer (PerkinElmer, Inc., Waltham, MA, USA). Powder X-ray diffraction (XRD) patterns of the sample was measured on a D/max 2550 diffractometer in the 5–40° region (2°/min) (Rigaku Corp., Tokyo, Japan). The HRMS mass spectral analysis was performed by a QStar Elite (Applied Biosystems SCIEX) mass spectrometer (AB Sciex, Toronto, Canada). Thermo-gravimetric analysis (TGA) was performed under N_2_ (Mettler-Toledo Inc., Columbus, OH, USA). Gas chromatography (GC) analysis was performed on GC-5890N(KEJIE INSTRUMENT, Nanjing, China). Inductively coupled plasma-atomic emission spectroscopy (ICP-AES) analysis was used on an IRIS Advantage 1000 instrument (Thermo Electron Corporation, Waltham, MA, USA). The scanning electron microscopy (SEM) analysis was performed on a model JSM-6700F scanning electron microscope (JEOL, Tokyo, Japan) and iridium (IXRF Systems) software with an accelerating voltage of 15 kV.

## 3. Results and Discussion

Synthetic routes of intermediates and corresponding supramolecular polymers were outlined in Schemes S1 and S2 and Scheme 1. The compounds, i.e., Salphen and BiSalphen, were prepared by Schiff base condensation between 1,2-diaminobenzene or 3,3-diaminobenzidine and salicylaldehyde. Their chemical structures were confirmed by ^1^H NMR and ^13^C NMR spectra (see Experimental Section and Supplementary Materials). The designed supramolecular polymers, denoted as BiCo-BiPy1:1 CSP and BiCo-BiPy1:2 CSP, were synthesized by a self-assembling of BiCo(Salphen) and 4,4-dipyridine in different stoichiometric ratios. Vigorous stirring of the mixtures led to the formation of polymeric precipitates, which were then filtrated and extracted with THF and methanol for 24 h to afford brownish powders (Scheme 1). The obtained polymers were insoluble in water and organic solvents (methanol, CHCl_3_ and *N*,*N*-dimethylformamide). To demonstrate the successful coordination between nitrogen atoms (pyridine ring) and cobalt ions (BiCo(Salphen)), two model reactions between Co(Salphen) and pyridine were conducted in tetrahydrofuran (THF) with two specific ratios (1:1 and 1:2) at 80 °C for 24 h. The obtained model products were denoted as Co(Salphen)@Py1:1 and Co(Salphen)@Py1:2), respectively (Scheme S2). The reaction solution was pipetted for high-resolution mass spectrum (HRMS) measurements. The respective species at m/z 475.31 and 532.16 corresponding to [M + Na]^1+^ and [M + H]^1+^ for Co(Salphen)@Py1:1 and Co(Salphen)@Py1:2 were detected, which demonstrated the feasibility of constructing supramolecular polymers by applying coordination chemistry in different ratios (Figures S2 and S3).

**Figure 1:**
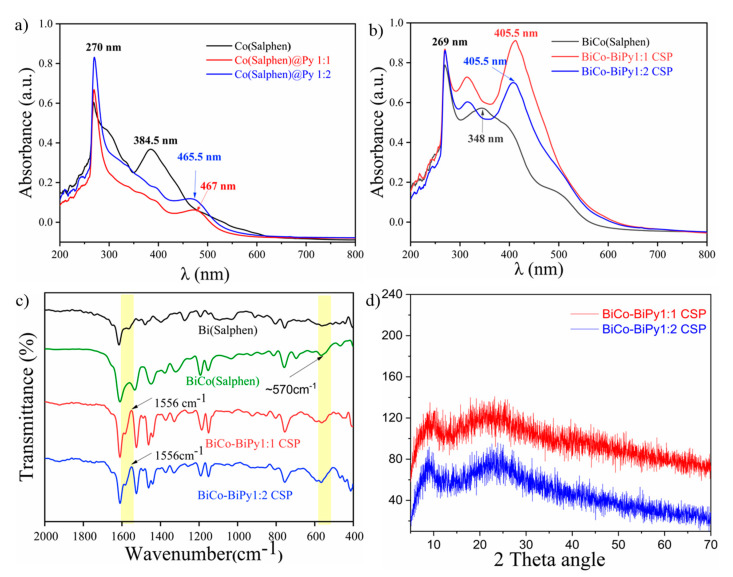
(**a**) UV-vis spectra of Co(Salphen), Co(Salphen)@Py1:1 and Co(Salphen)@Py1:2; (**b**) UV-vis spectra of BiCo-BiPy1:1 Co(Salphen) supramolecular polymers (CSP) and BiCo-BiPy1:2 CSP; (**c**) FT-IR spectra of Bi(Salphen), BiCo(Salphen), BiCo-BiPy1:1 CSP and BiCo-BiPy1:2 CSP; (**d**) XRD profiles of BiCo-BiPy1:1 CSP and BiCo-BiPy1:2 CSP.

Further structural characterizations of the obtained CSP were performed by employing UV-vis spectra and Fourier transform infrared (FT-IR) spectra. Figure 1a showed strong support for the model reactions. The adsorption peak at 270 nm can be assigned to a π–π* transition of the aromatic structures on Co(Salphen) [29,30], and the band centered at ~300 nm can be ascribed to the intraligand n–π* electronic transitions of the nonbonding electrons of the azomethine nitrogen atoms [31]. In addition, the signal centered at ~384.5 nm can be attributed to intramolecular metal–ligand interactions within the whole complex (meta–ligand d–π* charge transfer transitions (MLCT)) [29,30]. In the spectra of Co(Salphen)@1:1 (~466.5 nm) and Co(Salphen)@1:2 (~467 nm), the corresponding peaks exhibited an obvious red shift compared to that of Co(Salphen) (~384.5 nm), which confirmed the successful coordinations. Similarly, BiCo-BiPy1:1 CSP and BiCo-BiPy1:2 CSP showed a similar rule in UV-vis spectra, indicating the strong interactions between cobalt ions and nitrogen atoms of 4,4-bipyridine (Figure 1b). The FT-IR spectra displayed the typical new generated peak at ~570 cm^−1^, which was associated with the stretching vibrations of coordination bond between cobalt ions and C=N, suggesting that cobalt ions were coordinated with Salphen (Figure 1c). Similarly, the absorption peak at ~1556 cm^−1^ (assigned to the vibrational bands of C=N bonds of pyridine) verified the successful coordination between the cobalt ions and nitrogen atoms of 4,4-bipyridine. Additionally, powder X-ray diffraction (PXRD) measurement of BiCo-BiPy1:1 CSP and BiCo-BiPy1:2 CSP exhibited very broad peaks, suggesting a rather low degree of crystallinity of CSP (Figure 1d). In addition, the residual cobalt ions contents of BiCo-BiPy1:1 CSP and BiCo-BiPy1:2 CSP were detected to be at 13.09% and 11.23% using inductively coupled plasma-atomic emission spectroscopy measurements (ICP-AES) after fully degrading the samples in nitrohydrochloric acid, respectively. These results were consistent with the theoretical values (13.10% for BiCo-BiPy1:1 CSP and 11.15% for BiCo-BiPy1:2 CSP) according to the proposed chemical structures (Scheme 1).

**Figure 2:**
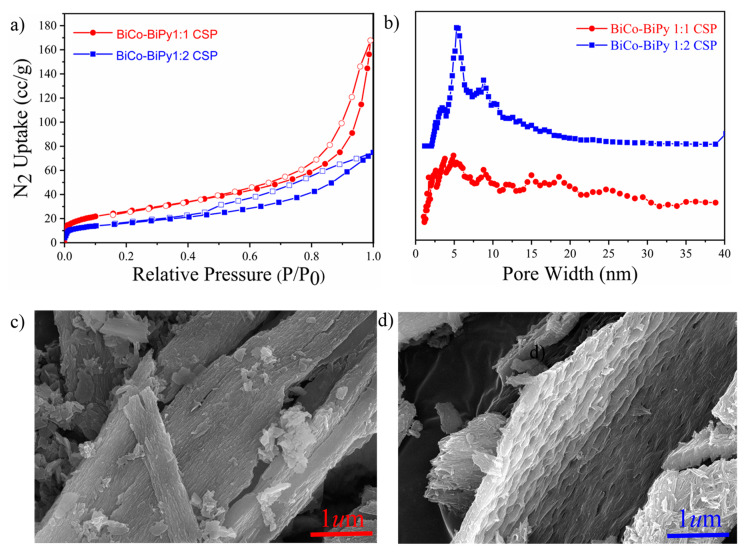
(**a**) Nitrogen adsorption (filled symbols)/desorption (open symbols) isotherms for BiCo-BiPy1:1 CSP and BiCo-BiPy1:2 CSP obtained at 77 K; (**b**) pore size distributions of BiCo-BiPy1:1 CSP and BiCo-BiPy1:2 CSP; (**c**,**d**) SEM images of BiCo-BiPy1:1 CSP and BiCo-BiPy1:2 CSP.

Porous properties of the supramolecular polymers were characterized by an N_2_ adsorption–desorption experiment at 77 K. Brunauer–Emmett–Teller (BET) surface areas of BiCo-BiPy1:1 CSP and BiCo-BiPy1:2 CSP were determined to be 95 and 58 m^2^ g^−1^, respectively. Their respective total pore volumes were calculated to be 0.26 and 0.12 cm^3^ g^−1^ at P/P_0_ = 0.99 (Figure 2a,b and Table S1). The porous structure of BiCo-BiPy1:1 with a higher BET surface area may result from the molecular chain twisting and intermolecular stacking effect, thus resulting in the formation of certain pores. It should be noted that the moderate BET surface areas and large pore volumes are beneficial for mass transfer of guest molecules. In particular, the high percentage of mesopores (>80%, Table S1) present in the designed samples offered shelters that sufficiently encapsulated oxygen and organic small molecules during the catalytic process, which allowed more efficient product mass transfer and substrate diffusion. The morphologies of supramolecular polymers were probed by scanning electron microscopy (SEM), and the images clearly exhibited sheet-like and plate-like structure, respectively (Figure 2c,d). Additionally, thermogravimetric analysis (TGA) of BiCo-BiPy1:1 CSP and BiCo-BiPy1:2 CSP showed that a slight mass loss was observed upon heating up to 200 °C under N_2_. The initial weight loss before 200 °C was attributed to the moisture trapped in the cavities, while the weight loss after 200 °C was attributed to the degradation of organic networks or cleavage of coordination bond. Compared to the precursor BiCo(Salphen), the designed supramolecular polymers BiCo-BiPy1:1 CSP and BiCo-BiPy1:2 CSP delivered higher stability (Figure S4), which may attribute to their rigid structures.

Given the robust and porous structure of the as-prepared polymers, their catalytic performance was explored using aerobic oxidation of TMP as a model reaction. The oxidation of TMP was generally conducted in relatively high temperatures and required the presence of H_2_O_2_ oxidant as an oxidant, while few works focus on using molecular oxygen as an oxidant (Table 1, Entries 1–8). Meanwhile, some catalysts are still suffering from low conversion and selectivity. Unlike in previous studies, in this work, the oxidation was conducted using O_2_ as a clean oxidant. The transformation enabled by BiCo-BiPy1:1 CSP gave a high conversion (>99%) and selectivity (>99%), which was significantly higher than that catalyzed by BiCo-BiPy1:2 CSP (conversion: 27%, selectivity: >99%) (Table 1, Entries 9 and 10). The catalytic activity expressed by a turnover number (TON) value was higher for BiCo-BiPy1:1 CSP (22.5) than the TON values found for BiCo-BiPy1:2 CSP (7.2) (Table 1, Entries 9 and 10). Furthermore, the turnover frequency (TOF) value for BiCo-BiPy1:1 CSP (1.88 h^-1^) is larger than that for BiCo-BiPy1:2 CSP (0.6 h^−1^) (Table 1, Entries 9 and 10). The low catalytic activity in the latter case may attribute to steric hindrance of BiCo-BiPy1:2 CSP, which hinders oxygen from penetrating through the polymer skeleton to activate O_2_, resulting in generating less activated oxygen species [32]. Although the cobalt ions in BiCo-BiPy1:1 CSP are coordinated with the *N* atom of pyridine, the less steric hindrance of BiCo-BiPy1:1 CSP allows the cobalt to be fully exposed to oxygen, which allows BiCo-BiPy1:1 CSP sufficient contact with molecular oxygen and then renders the molecular oxygen to be activated. Additionally, BET surface areas may also play a significant role in the catalytic process. Compared with BiCo-BiPy1:2 CSP, BiCo-BiPy1:1 CSP with higher BET surface areas and larger pore volumes appears to be more effective for mass transfer of guest molecule O_2_, which could generate more activated oxygen species [33,34]. No TMBQ was observed in the case without adding the supramolecular polymers, demonstrating the indispensable role of the catalyst in oxidation of TMP (Table 1, Entry 11). Almost no product was detected for the catalytic reactions in the absence of oxygen (Table 1, Entry 12), which verified the critical role of oxygen in the catalytic process. Furthermore, a survey of reaction conditions revealed that a high conversion (>99%) and an excellent selectivity (>99%) could be achieved in just 1.0 h under high pressure of O_2_ (1.0 MPa) (Table 1, Entry 13). Moreover, the corresponding Co(Salphen) supramolecular polymers resulted in the comparable reactivity compared to their homogeneous counterparts (Table 1, Entries 14–17). 

**Figure 3:**
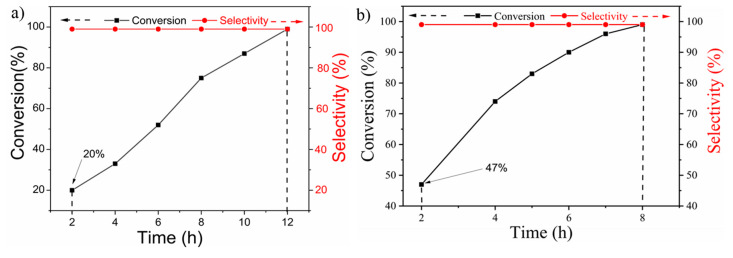
(**a**) Reaction kinetic curve: Reaction condition: TMP (68.1 mg, 0.5 mmol), methanol (2.0 mL), BiCo-BiPy1:1 CSP (10.0 mg), O_2_ (0.1 MPa) and 12 h; (**b**) reaction kinetic curve. Reaction conditions: BiCo-BiPy1:1 CSP (10.0 mg) was exposed to oxygen atmosphere for 6 h, TMP (68.1 mg, 0.5mmol), methanol (2.0 mL), BiCo-BiPy1:1 CSP (10.0 mg), O_2_ (0.1 MPa) and 8 h.

Furthermore, the reaction kinetics of the catalytic process were explored by using BiCo-BiPy1:1 CSP as the catalyst under 30 °C and atmospheric pressure. Notably, the conversion rate of TMP in the first 6 h was significantly lower than that in the later 6 h, suggesting that activated oxygen species played a critical role (Figure 3a). In the first 6 h, insufficient contact between catalyst and oxygen led to less reactive oxygen species, and the conversion rate of TMP was low. As reaction time prolonged, especially over the course of the later 6 h, the catalyst BiCo-BiPy1:1 CSP was sufficiently exposed to oxygen molecules to generate activated oxygen species Co-superoxo complex subsequently, with the conversion rate of TMP significantly higher compared to that in the first 6 h [39,40]. To validate this assumption, BiCo-BiPy1:1 CSP was pre-treated under oxygen atmosphere for 6 h. In the first 2 h, the oxidation of TMP in the presence of pre-treated BiCo-BiPy1:1 CSP reached 47% conversion, which was more than two-fold faster than that catalyzed by the unpretreated BiCo-BiPy1:1 CSP (conversion: 20%). Moreover, the reaction catalyzed by pre-treated BiCo-BiPy1:1 CSP (8 h) was significantly more accelerated than that of unpretreated BiCo-BiPy1:1 CSP (12 h), shortening the reaction time effectively (Figure 3b). These indicate that the activated oxygen species Co-superoxo complex can be a significant factor during the catalytic process [39,40].

**Figure 4:**
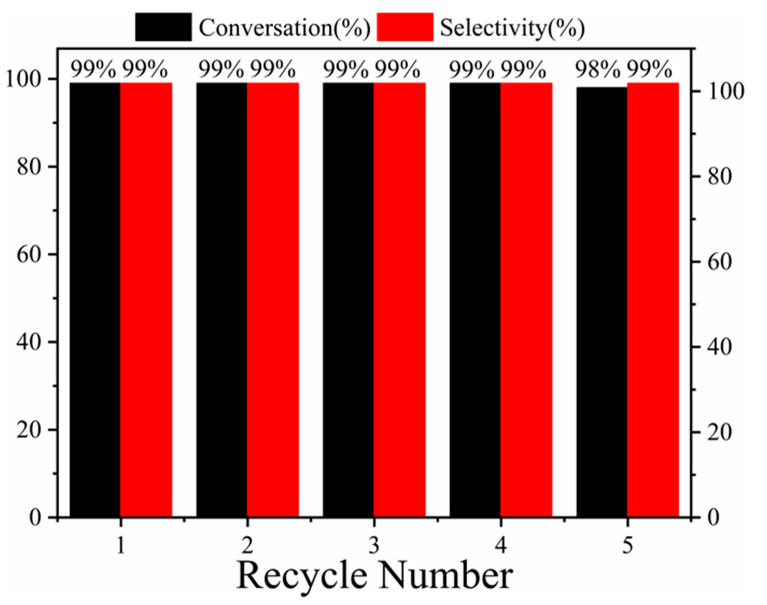
Cycling performance of BiCo-BiPy1:1 CSP for aerobic oxidation of TMP. Reaction conditions: TMP (68.1 mg, 0.5 mmol), methanol (2.0 mL), BiCo-BiPy1:1 CSP (10.0 mg), O_2_ (0.1 MPa) and 12 h.

Except for the conversion and selectivity, the recyclability of catalyst was another significant factor to evaluate the overall performance. Recycling experiments were performed by applying BiCo-BiPy1:1 CSP. As shown in Figure 4, BiCo-BiPy1:1 CSP could be recycled at least five times without a substantial catalytic efficiency decline. Significantly, the conversion (>99%) and selectivity (>99%) were retained in each cycle, validating the high stability of BiCo-BiPy1:1 CSP. These results demonstrated the reusability of the designed BiCo-BiPy1:1 CSP as heterogeneous catalysts in highly selective oxidation of TMP. Meanwhile, the designed BiCo-BiPy1:1 CSP towards selective oxidation of TMP to TMBQ delivered a great potential in large-scale application for the production cost for 1.0 g of TMBQ, which is only 2.93 CNY (TMP: 2.0 CNY/g) (Table S2).

In addition, to evaluate the cobalt leaching of BiCo-BiPy1:1 CSP, the reaction mixture was analyzed after 12 h. Surprisingly, no obvious change in either the conversion or the selectivity (Figure 4) was observed, and the cobalt content was <0.1 ppm in the filtrate as observed by ICP-AES, suggesting negligible cobalt leaching in the catalytic process. Furthermore, the TON value (22.7) was almost completely retained after five cycles. It may be concluded that the robust skeleton of BiCo-BiPy1:1 CSP offered a scaffold for cobalt, which effectively protected cobalt from leaching into the solution during the catalytic process. 

## 4. Conclusions

In summary, we have developed a simple and low-cost synthetic strategy to prepare Co(Salphen) supramolecular polymers for selective oxidation of TMP to TMBQ with oxygen as a clean oxidant. The obtained supramolecular polymer (BiCo-BiPy1:1 CSP), as an efficient heterogeneous catalyst, exhibited high conversion (>99%) and excellent selectivity (>99%) toward oxidation of TMP with nearly quantitative yields under a mild reaction condition. The results acquired in this work clearly demonstrate that the activated oxygen species Co-superoxo complex plays a critical role in the reactions. Presumably, the higher specific surface area and larger pore volumes of BiCo-BiPy1:1 CSP increase its pore accessibility to the substrates and thus enhances catalytic efficiency. Moreover, the heterogeneous catalyst could be recycled and reused at least five times. These findings allow us to view them as prospective heterogeneous catalysts for the clean and sustainable oxidation of TMP.

## Figures and Tables

**Table 1:**
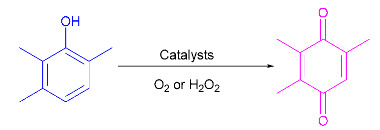
Different systems in oxidation of 2,3,6-trimethylphenol.

Entry	Catalyst	Oxidant	*T*/°C	Con/%	Sel/%	Time	TOF	TON
1^a^	Ti,Si-catalysts (A1) [12]	H_2_O_2_	80	99	96	18 min	4.6 min^−1^	82.8
2^a^	V_2_-POM/N-CNTs [15]	H_2_O_2_	60	100	99	15 min	-	-
3^a^	TiSBA-15(6) [19]	H_2_O_2_	80	99	97	60 min	3.0 min^−1^	180
4^b^	V_2_-POM [35]	H_2_O_2_	60	100	99	15 min	-	-
5^a^	Ti-HMS [36]	H_2_O_2_	60	97	84	6 h	-	-
6^a^	Au/TiO_2_ [36]	H_2_O_2_	60	96	0	6 h	-	-
7^a^	Ti/SiO_2_ (A) (1.97) [37]	H_2_O_2_	80	100	96	30 min	2.0 min^−1^	60
8^a^	Ti/SiO_2_ Davisil C [38]	H_2_O_2_	80	100	99	30 min	1.8 min^−1^	54
9^a, c^	BiCo-BiPy1:1 CSP	O_2_	30	>99	>99	12 h	1.88 h^−1^	22.5
10^a, c^	BiCo-BiPy1:2 CSP	O_2_	30	27	>99	12 h	0.60 h^−1^	7.2
11^a, c^	No catalyst	O_2_	30	<1	-	12 h	-	-
12^a^	BiCo-BiPy1:1 CSP	N_2_	30	<1	-	12 h	-	-
13^a, d^	BiCo-BiPy1:1 CSP	O_2_	30	>99	>99	1.0 h	22.5 h^−1^	22.5
14^a, c^	Co(Salphen)Py1:1	O_2_	30	>99	>99	12 h	1.88 h^−1^	22.6
15^b, c^	Co(Salphen)Py 1:2	O_2_	30	32	>99	12 h	0.71 h^−1^	8.5
16 ^b, c^	BiCo(Salphen)	O_2_	30	>99	>99	12 h	1.55 h^−1^	18.6
17^b, c^	Co(Salphen)	O_2_	30	>99	>99	12 h	1.55 h^−1^	18.7

^a^ heterogeneous; ^b^ homogeneous; ^c^ Reaction condition: 2,3,6-trimethylphenol (TMP) (68.1 mg, 0.5 mmol), catalyst (10.0 mg), methanol (2.0 mL), O_2_ (0.1 MPa) and 12 h; ^d^ Reaction condition: TMP (68.1 mg, 0.5 mmol), catalyst (10.0 mg), methanol (2.0 mL), O_2_ (1.0 MPa) and 1.0 h.

## Supplementary Materials

The following are available online at https://www.mdpi.com/2073-4360/12/5/1076/s1.
