# Supplementary material for: Stable Non-Covalent Co(Salphen)-Based Polymeric Catalyst for Highly Efficient and Selective Oxidation of 2,3,6-Trimethylphenol
Source: Polymers (Basel). 2020 May 8;12(5):1076. doi: 10.3390/polym12051076 (PMC7285139; doi:10.3390/polym12051076)
Supplement: Supplementary file 1 [file polymers-12-01076-s001.pdf]

Supplementary Materials

Stable non-covalent Co(Salphen) based polymeric catalyst for highly efficient and selective oxidation of 2,3,6-trimethylphenol

Weijie Zhang, Lingling Hu, He Zhang, Chunyue Pan and Juntao Tang**

**Corresponding author: Chunyue Pan (E-mail: panchunyue@csu.edu.cn) and Juntao Tang (E-mail: reynardtang@csu.edu.cn)*

Postal address: College of Chemistry and Chemical Engineering, Central South University, 932 South Lushan Road, Changsha 410083, Hunan, P. R. China.

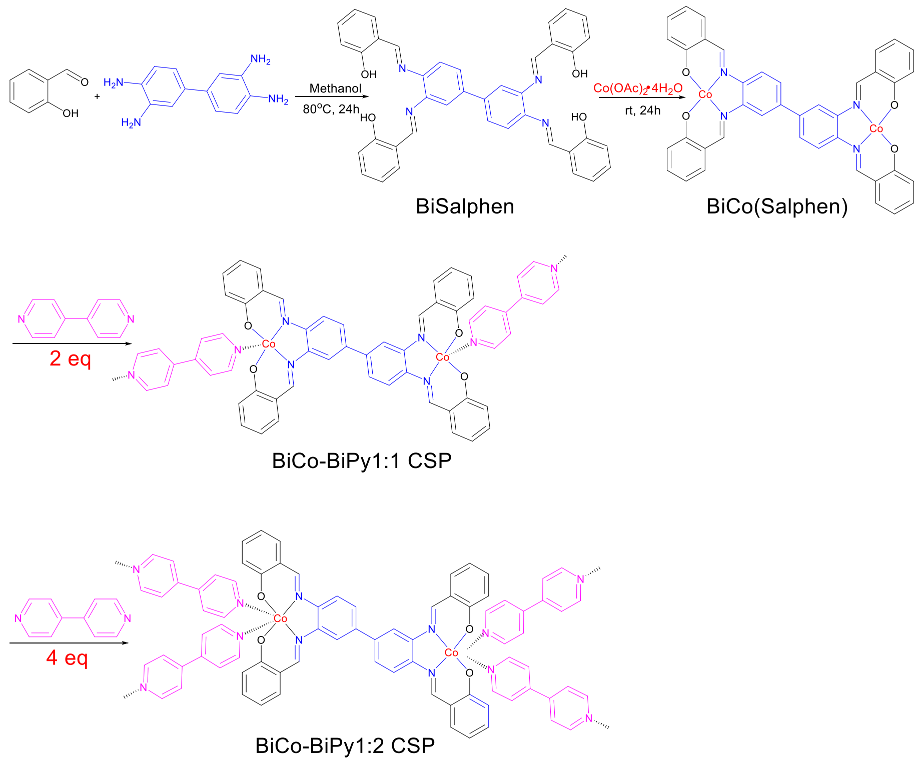

Scheme S1. Synthetic route of Bi[Co(Salphen)], BiCo-BiPy1:1 and CSP BiCo-BiPy1:1 CSP

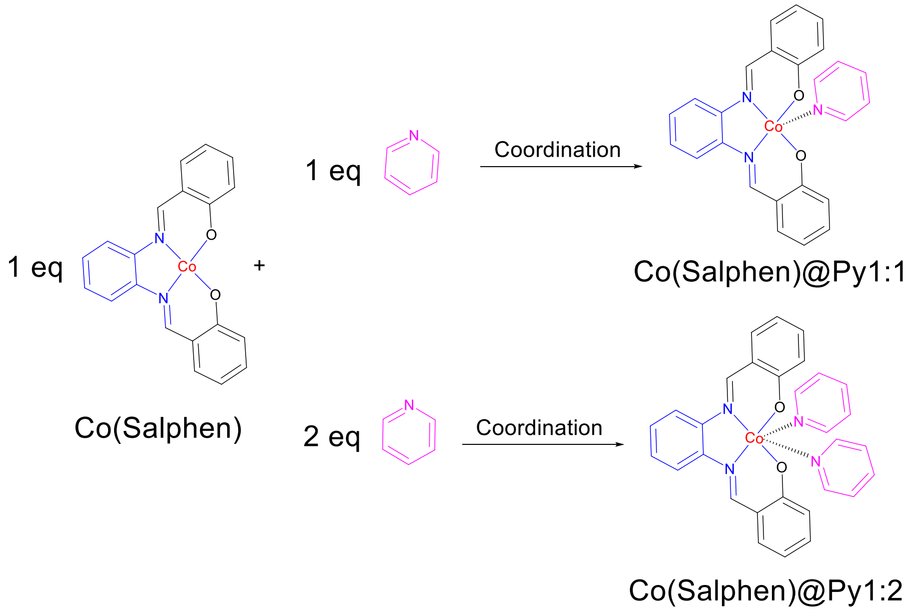

Scheme S2. Model reactions between Co(Salphen) and pyridine

Procedure:

Preparation of ***Salphen***

In a three-necked round-bottom flask equipped with a constant pressure dropping funnel, a solution of 1,2-diaminobenzene (1.73 g, 16 mmol) in methanol (50 mL) was stirred at 80 °C for 10 min. Subsequently, salicylaldehyde (4.1 g, 33.9 mmol) in methanol (50 mL) was dropped into the above mixture, and the reaction flask was kept stirring for 12 h at 80 °C under N_2. After that, the mixture was cooled under 0 °C for 12 h and the resulting precipitate was collected by filtration, washed with methanol and dried to afford the desired product as a yellow solid (5.1 g, yield 88%). ^{1}H NMR ($CDCl_3$, 400 MHz): δ 13.06 (s, 2H),8.65 (s, 2H), 7.41-7.36 (m, 6H), 7.26-7.24 (m, 2H), 7.07-7.05 (d, 2H) and 6.96-6.94 (t, 2H) ppm. ^{13}C NMR ($CDCl_3$, 100 MHz): δ 163.7, 161.4, 142.6, 133.4, 132.3, 127.7, 119.9, 119.2, 119.0, 117.6,120.2, 119.1, 118.4 and 117.6 ppm.

Preparation of ***Co(Salphen)***

To a solution of $Co(OAc)_2 \cdot 4H_2O$ (4.8 g, 19.3 mmol) in methanol (100 mL), Salphen (5.0 g, 15.8 mmol) was added under N_2 atmosphere. The above mixture was stirred at 80 °C for 12 h. After cooling to room temperature, the product CoSalphen was collected by filtration (7.0 g, 78%).

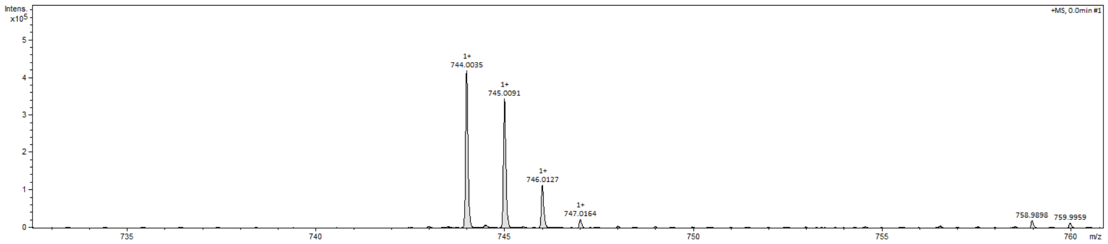

Figure. S1. HRMS of Bi[Co(Salphen)]

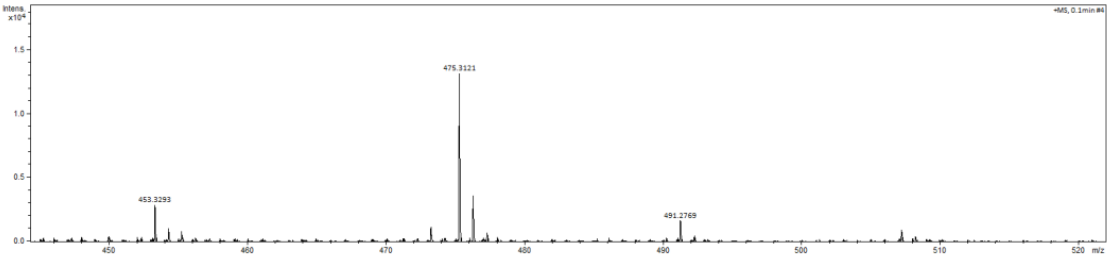

Figure. S2. HRMS of Co(Salphen)@Py 1:1

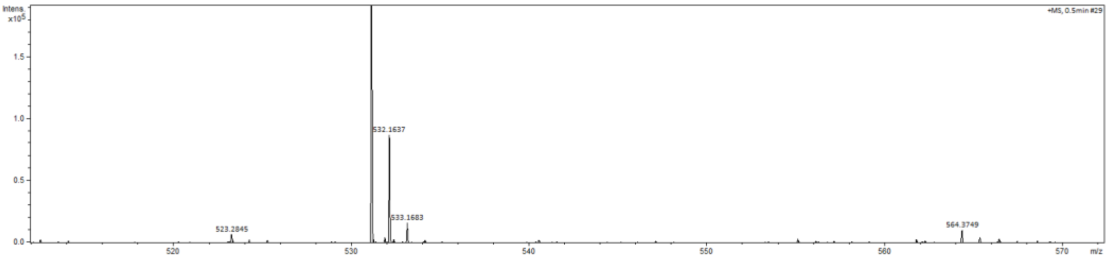

Figure. S3. HRMS of Co(Salphen)@Py 1:2

Table S1. Porosity parameters of BiCo@BiPy

Sample	S_{BET}^{a}	V_{total}^{b}	V_{micro}	V_{micro}/V_{total}	D_{pore}^{c} nm
BiCo@BiPy 1:1	95	0.259	0.0338	0.13	8
BiCo@BiPy 1:4	58	0.116	0.0217	0.18	11

[a]Brunauer-Emmett-Teller surface area in m^2 g^{-1}. [b]Pore volume determined from the N_2 isotherm at P/P_0=0.99 in cm^3 g^{-1}. [c]Pore size derived from N_2 isotherm with the NLDFT approach.

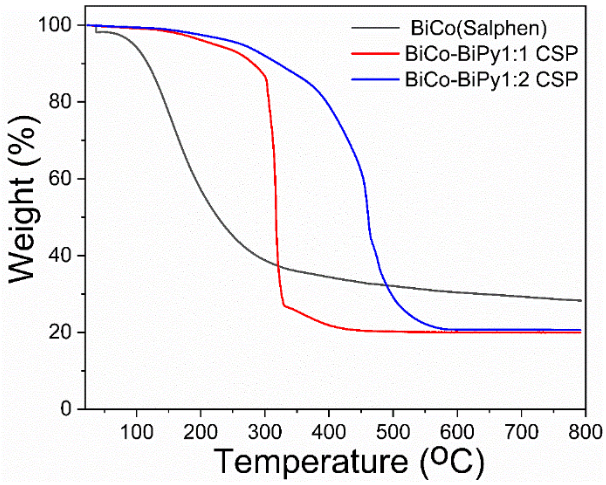

Figure. S4. TGA of BiCo(Salphen), BiCo@BiPy 1:1 and BiCo@BiPy 1:2

Table S2. Materials quantities and cost for the synthesis of trimethyl-1,4-benzoquinone

Chemical name	Weight of reagent (g or L)	Price (yuan/g or L)	Cost (RMB yuan)
[1,1'-biphenyl]-3,3',4,4'-tetraamine	1.0 g	2.0	2.0
THF	0.1	15	1.5
Methanol	0.25 L	4.0	0.1
Salicylaldehyde	0.1g	2.74	0.27
BiSalphen (yield: 91%)	0.25 g	1.44	0.36
Methanol	0.03 L	4.0	0.12
THF	0.1 L	15	1.5
$Co(OAc)_2{\cdot}4H_2O$	0.24 g	1.5	0.36
BiCo(Salphen) (yield: 89%)	0.1 g	2.23	0.223
THF	0.05 L	15	0.75
4,4-dipyridine	0.02 g	0.15	0.0032
DMF	0.15 L	8	1.2
Methanol	0.05 L	4.0	0.2
BiCo-BiPy1:1 CSP (yield: 51%)	0.01g	36.3	0.36
2,3,6-trimethylphenol	0.0681	2.0	0.14
Methanol	0.002	4.0	0.008
2,3,5-trimethylcyclohexa-2,5-diene-1,4-dione	N/A	2.93	N/A

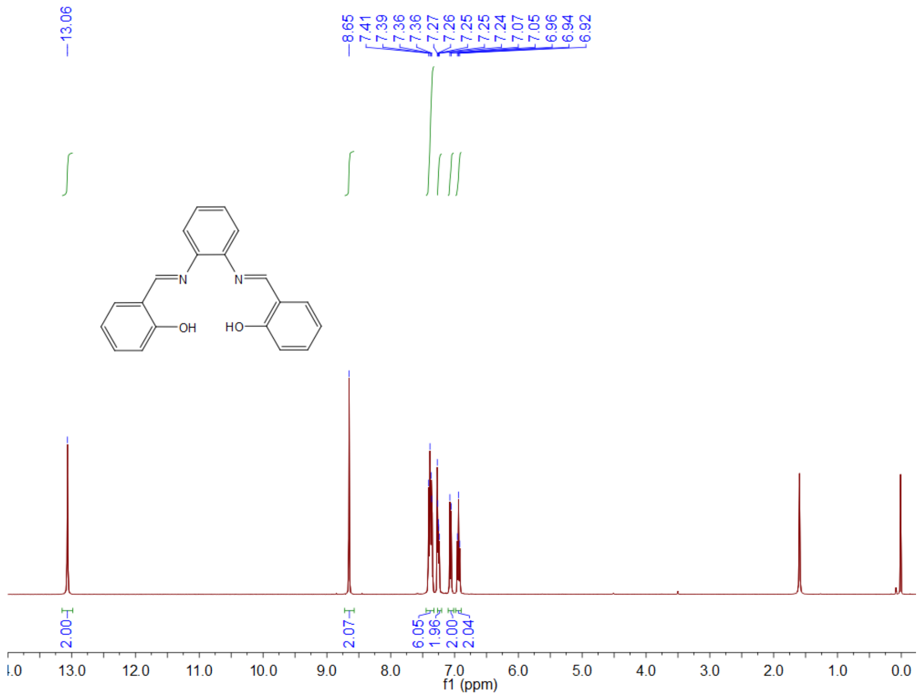

^{1}H NMR of Salphen

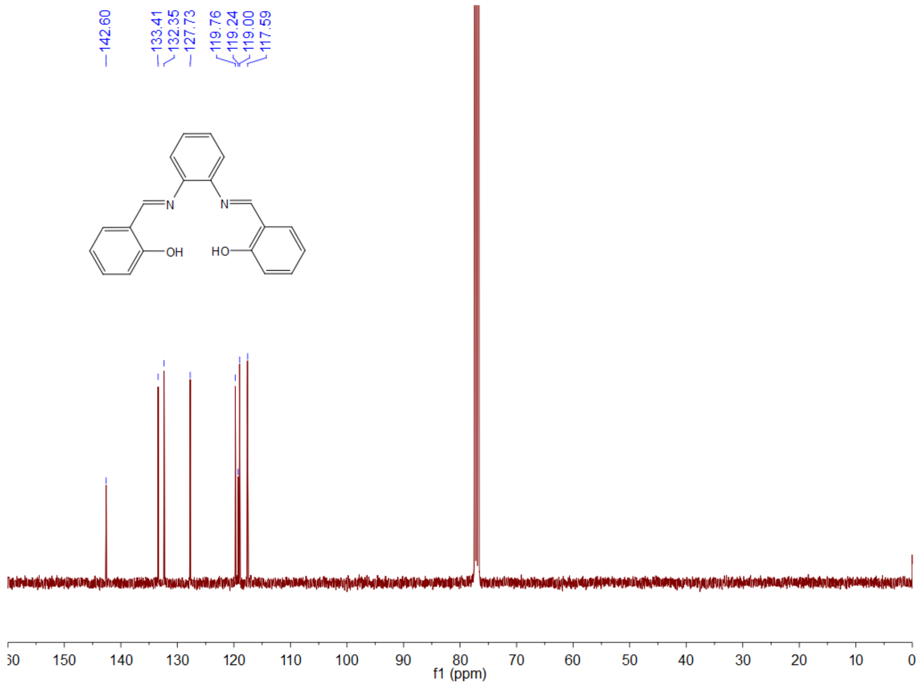

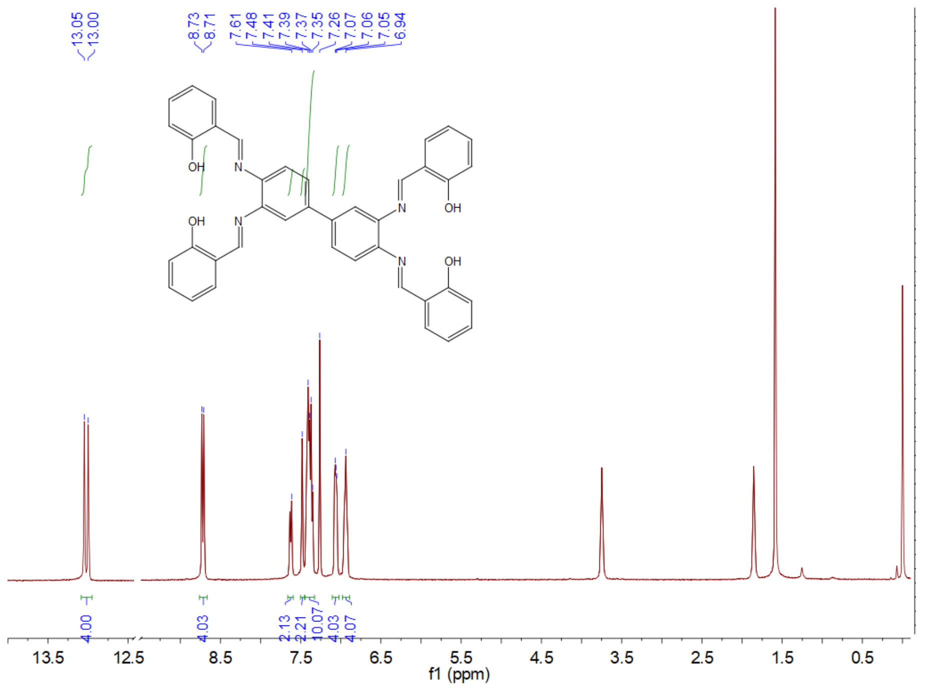

^{1}H NMR of BiSalphen

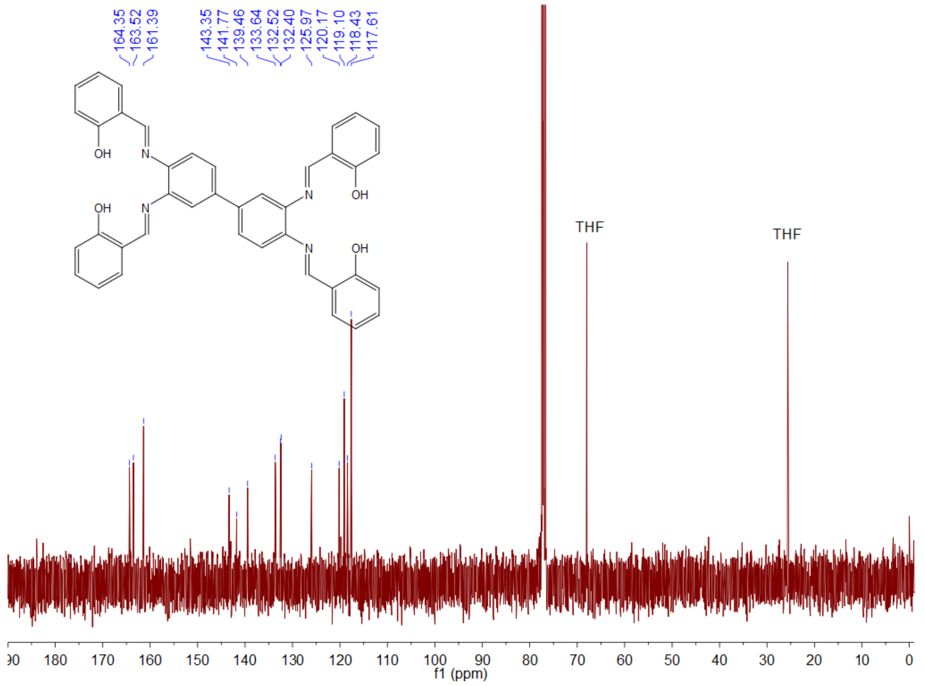

^{13}C NMR spectra of BiSalphen

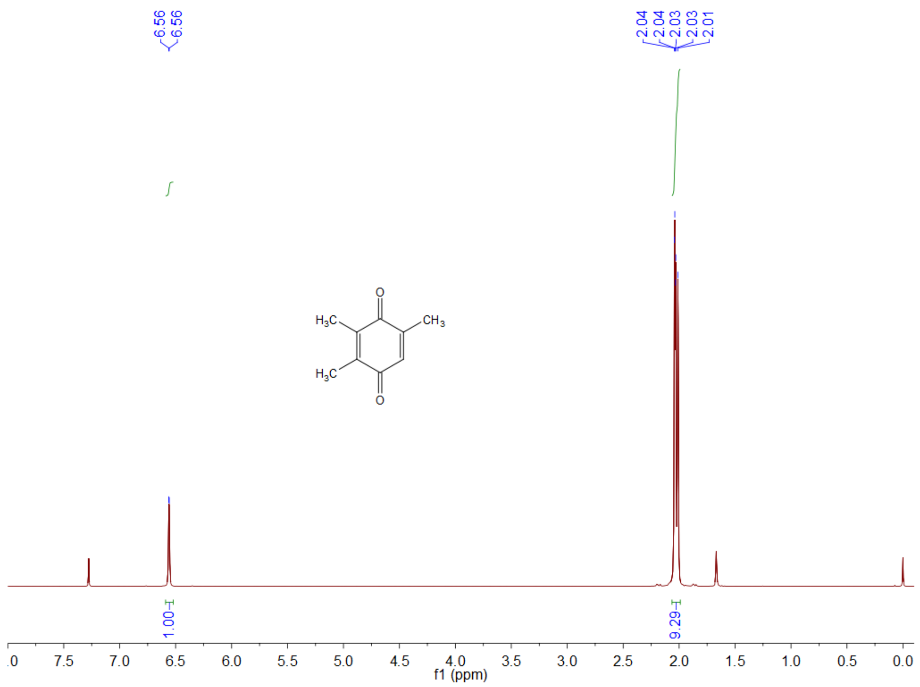

^{1}H NMR of trimethyl-1,4-benzoquinone

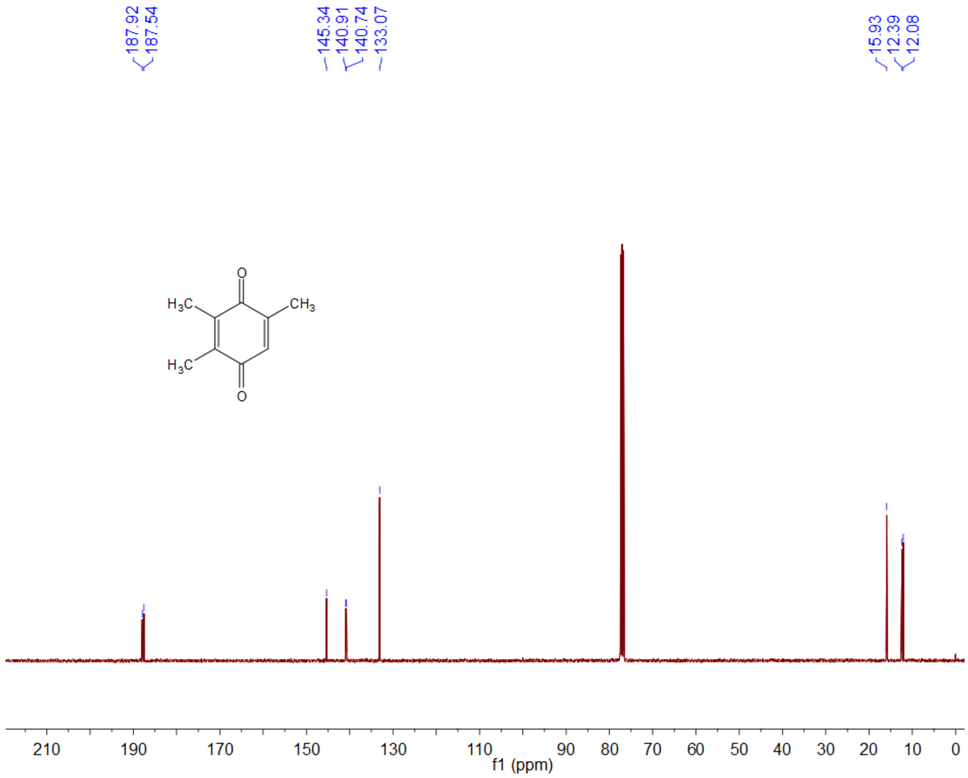

^{13}C NMR of trimethyl-1,4-benzoquinone

Trimethyl-1,4-benzoquinone: ^{1}H NMR ($CDCl_3$, 400 MHz): δ 6.56 (s, 1H) and 2.04-2.01 (m, 9H) ppm. ^{13}C NMR ($CDCl_3$, 100 MHz): δ 187.9, 187.5, 145.3, 140.9, 140.7, 133.0, 15.9, 12.4 and 12.1 ppm.
